# Supplementary material for: Machine learning algorithm-based biomarker exploration and validation of mitochondria-related diagnostic genes in osteoarthritis
Source: PeerJ. 2024 Sep 10;12:e17963. doi: 10.7717/peerj.17963 (PMC11397131; doi:10.7717/peerj.17963)
Supplement: Supplemental Information 2 [file peerj-12-17963-s002.docx]

Gapdh-F: AGGTCGGTGTGAACGGATTTG

Gapdh-R: GGGGTCGTTGATGGCAACA

Grpel1-F: TTGGCACTGTCGTTCAGGC

Grpel1-R: GGATCTGTCTTTGGCTCACAAT

Ifngr2-F：TCCTCGCCAGACTCGTTTTC

Ifngr2-R：ACGGCTCCCAAGTTAGAATCT

Adamts5-F：CCCAGGATAAAACCAGGCAG

Adamts5-R：CGGCCAAGGGTTGTAAATGG

Mmp13-F: TGTTTGCAGAGCACTACTTGAA

Mmp13-R: CAGTCACCTCTAAGCCAAAGAAA

Aggrecan-F: GTGGAGCCGTGTTTCCAAG

Aggrecan-R: AGATGCTGTTGACTCGAACCT

Col2a1-F: GGGTCACAGAGGTTACCCAG

Col2a1-R: ACCAGGGGAACCACTCTCAC
