# Supplementary figures and images for: Machine learning algorithm-based biomarker exploration and validation of mitochondria-related diagnostic genes in osteoarthritis
Source: PeerJ. 2024 Sep 10;12:e17963. doi: 10.7717/peerj.17963 (PMC11397131; doi:10.7717/peerj.17963)

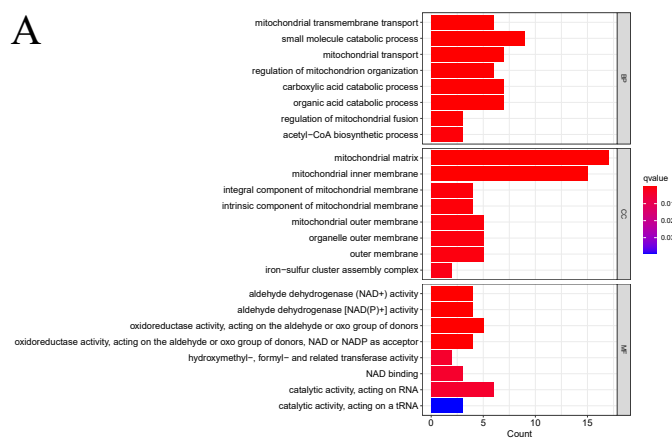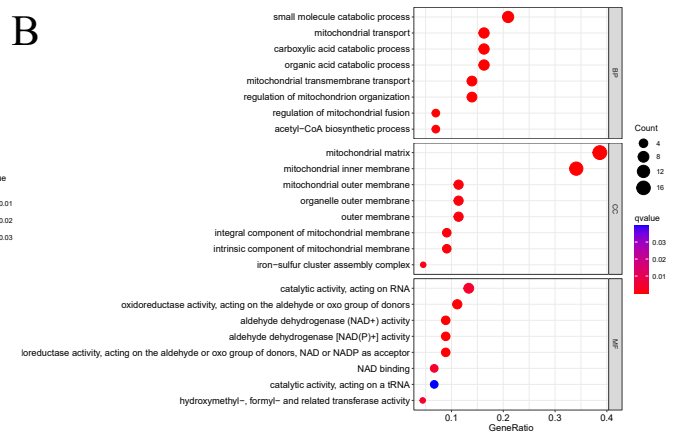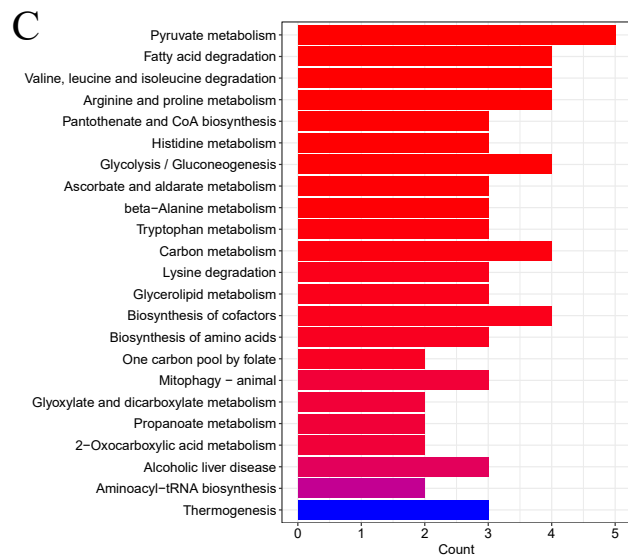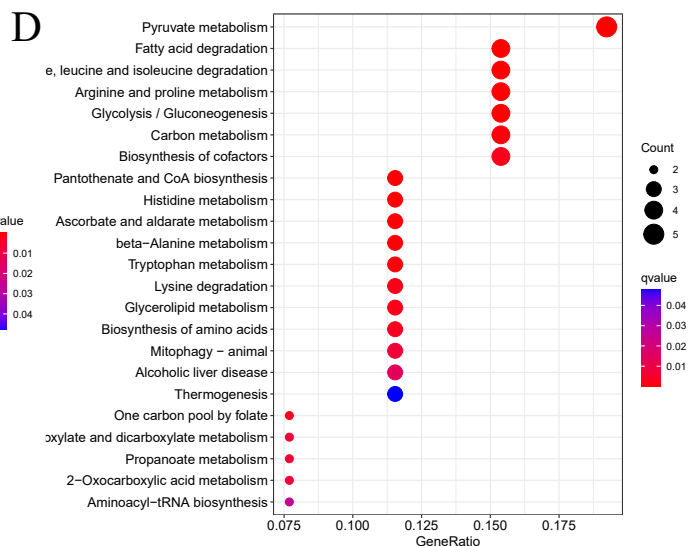

Supplement: Supplemental Information 4 — GO and KEGG enrichment analysis results of differentially expressed MRGs. (A): Bar chart for GO analysis results; (B): Bubble chart for GO analysis results; (C): Bar chart for KEGG analysis results; (D): Bubble chart for KEGG analysis results. [file peerj-12-17963-s004.pdf]

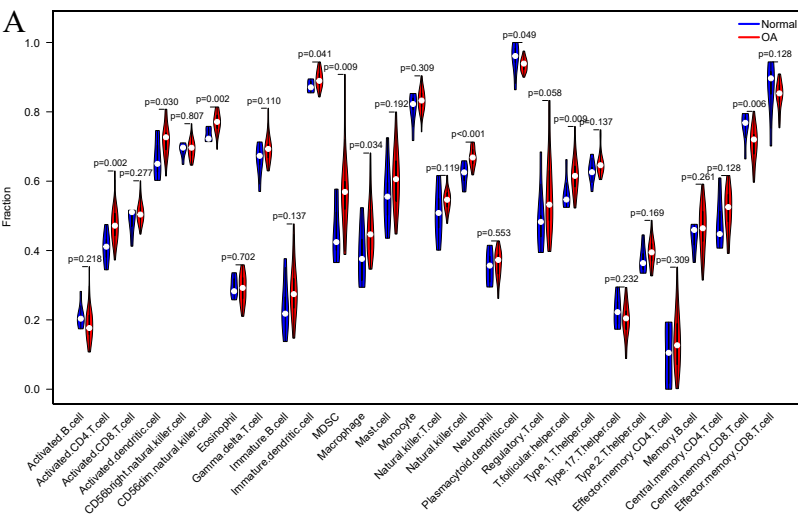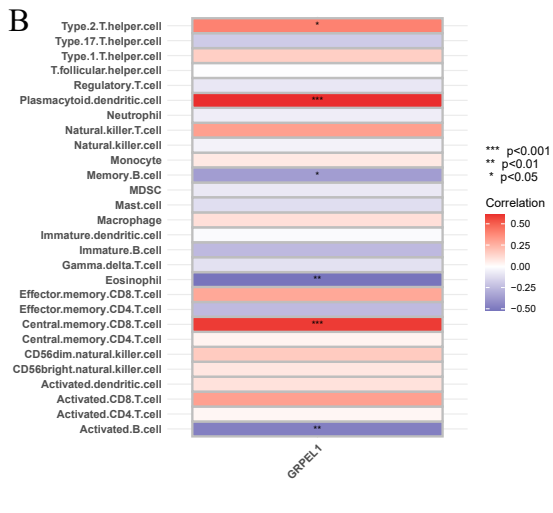

Supplement: Supplemental Information 5 — Immune cell infiltration analysis based on the validation dataset GSE57218. (A): Differences in immune cell infiltration of 28 immune cells between OA and normal samples; (B): Correlation between GRPEL1 and abundance of immune cell infiltration. [file peerj-12-17963-s005.pdf]
